# A qualitative study of factors related to cardiometabolic risk in rural men

**DOI:** 10.1186/s12889-016-2977-1

**Published:** 2016-04-11

**Authors:** Emily H. Morgan, Meredith L. Graham, Sara C. Folta, Rebecca A. Seguin

**Affiliations:** Division of Nutritional Sciences, Cornell University, Savage Hall, Ithaca, NY 14853 USA; Friedman School of Nutrition Science and Policy, Tufts University, 150 Harrison Avenue, Boston, MA 02111 USA

**Keywords:** Men’s health, Rural health, Cardiometabolic disorder, Cardiovascular disease, Diabetes, Prevention, Qualitative research

## Abstract

**Background:**

Rural men are known to have poor health behaviors, which contribute to their elevated burden of cardiometabolic disorders in the United States. Although regular physical activity, healthy eating, and avoiding tobacco can reduce cardiometabolic risk, little is known about how to engage rural men in health promotion programs. To bridge this gap in evidence, we investigate knowledge of modifiable cardiometabolic risk factors among rural men in the western United States, identify their concerns related to heart health and motivation to reduce risk, and explore individual, social, and community-level influences on heart-healthy behaviors, specifically diet, physical activity, and tobacco use.

**Methods:**

We conducted seven focus groups with 54 sedentary, overweight/obese men (mean body mass index [BMI] = 31.3 ± 4.6) aged 43–88 residing in government-designated “medically underserved” rural Montana towns in September and October 2014. All sessions were audio-recorded and transcribed verbatim. Transcripts were coded and analyzed thematically using Nvivo software. Participants also completed a brief questionnaire about personal characteristics and health behaviors. These data were explored descriptively.

**Results:**

Despite being classified as overweight/obese and sedentary, no participants reported to be in poor health. Many men described health relative to self-reliance and the ability to participate in outdoor recreation; concern with health appeared to be related to age. Participants were generally knowledgeable of heart-healthy behaviors, but many felt fatalistic about their own risk. Catalysts for behavior change included a serious medical event in the household and desire to reduce aging-associated functional decline. Barriers to adopting and maintaining healthy eating and physical activity habits and abstaining from tobacco included normative beliefs around masculinity and individual liberty, the limited social universe of small towns, winter weather, time constraints, and preferences for unhealthy foods. Facilitators included behavioral self-monitoring, exercising with a partner, and opportunities for preferred activities, such as hunting and team sports.

**Conclusions:**

These findings provide important insight about influences on rural men’s health behaviors and provide guidance for possible intervention strategies to promote cardiometabolic health.

**Trial registration:**

ClinicalTrials.gov NCT02499731. Registered 1 July 2015.

## Background

Cardiometabolic disorders, including cardiovascular disease and type two diabetes, are the leading causes of disability and death globally. In the U.S., approximately one in every three adults has cardiovascular disease [1] and more than one in ten has diabetes [2]. Persons living in rural areas in the U.S. are more likely to be diagnosed with these diseases than persons living in urban areas. Data from the U.S. Centers for Disease Control and Prevention’s (CDC’s) 2008 Behavioral Risk Factor Surveillance System revealed that prevalence rates of coronary heart disease – the most common form of cardiovascular disease – and diabetes were 38.8 % and 8.6 % higher among rural respondents compared to urban respondents [3].

Geographic inequalities in health have widened in recent decades [4–6] and represent an important public health challenge. For instance, in the last half century, residents in urban areas experienced faster declines in mortality and larger gains in life expectancy than those in rural areas [4, 5]. Prevalence of risk factors for cardiometabolic disorders, including obesity [7], physical inactivity [8], poor dietary habits [7, 9], and tobacco use [10, 11], tend to be higher in rural areas. Cardiometabolic risks may be further elevated among rural men. Compared to women, men tend to have lower cardiovascular knowledge and perception of risk [12], are more likely to be overweight or obese [13] yet consider themselves to be healthy weight [14], and are more likely to engage in a range of behaviors that may adversely affect cardiometabolic health [15].

Evaluation of successful health behavior change interventions has provided evidence on effective strategies to improve physical activity levels, dietary habits, and tobacco behaviors; yet men have been underrepresented in the research [16–18] and the lessons learned may not, therefore, be generalizable to them. To date, there has been little research addressing how to engage rural men in chronic disease prevention programs, and the evidence that does exist for U.S. populations focuses mainly on African American and Latino men in the South or Northwest [19, 20]. Little is known about how best to engage those living in other regions of the country and this gap in the literature hinders the development of effective interventions.

The purpose of this study was to determine knowledge of modifiable cardiometabolic risk factors among men living in Montana, identify their concerns related to heart health and motivation to reduce risk, and explore individual, social, and community-level influences on heart-healthy behaviors, specifically diet, physical activity, and tobacco use. Montana is the fourth largest state by area in the U.S. and one of the most rural. The most recent U.S. Census documented 6.8 persons per square mile compared to the national average of 87.4, with 44.1 % of residents living in rural areas (clusters <2500 people) [21]. The state’s poverty rate is comparable with the national average (16.5 % compared to 15.8 %) [22] and approximately one in eight adults works in natural resources, construction, or maintenance occupations [23]. The long-term goal of this study is to inform the development of a cardiovascular disease and diabetes prevention program specifically targeting rural men.

## Methods

We conducted seven focus groups in six government-designated medically underserved [24] rural Montana communities (average population <2000, Fig. 1) in September and October 2014. These data were collected as part of formative research for the Strong Hearts, Healthy Communities trial. This trial aims to reduce cardiovascular disease, improve quality of life, and reduce cardiovascular disease-related health care costs in rural communities [25].

**Fig. 1:**
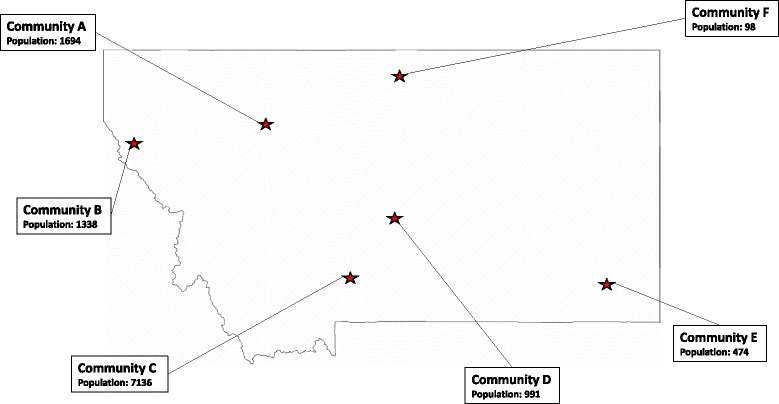
Geographic representation of the study sample. This figure shows the location and population of the rural communities in Montana where focus groups were conducted in September and October 2014

At each site, National Institute of Food and Agriculture (NIFA) Extension agents recruited a purposive sample of overweight, sedentary men aged 40 and older, using a variety of community-based strategies, including press releases, flyers, and website posts. We determined eligibility using a brief screening tool that asked men to self-report age, height, weight, and activity-level. We defined sedentary as participating in no more than one bout of 30 min (or more) of physical activity per week, on average, over the past three months. Focus groups were held at local community sites and stratified by age (40–64, 65+).

An experienced focus group facilitator (MLG) led the sessions, which lasted between 60 and 90 min. The discussion guide was informed by the socio-ecological model and developed by MLG, SCF, and RAS (public health researchers with extensive qualitative methodology experience). The questions explored awareness and knowledge about factors related to cardiometabolic risk; access to health care services and information; attitudes, perceptions, barriers, and facilitators to heart-healthy behaviors; and understanding community in a rural environment. We conducted a pilot focus group in Ithaca, NY and subsequently refined the guide to improve question comprehension and discussion flow. In addition to participating in the discussion, we asked participants to complete a brief questionnaire that asked about demographic characteristics and health behaviors. Participants received $50 compensation. The Institutional Review Board at Cornell University approved all protocols and materials (Protocol # 1402004505) and all participants provided written consent to take part.

Focus groups were transcribed verbatim and EHM reviewed transcripts against audio-files for accuracy. All transcripts were imported into NVivo version 10 (QSR International Pty Ltd) and coded by question. We developed an initial descriptive coding framework based on the main focus group topics and themes that emerged during transcript reviews. Two people independently coded a subset of the data and reviewed coding decisions line-by-line. Inter-rater reliability was high, with observed agreement >95 % and prevalence- and bias-adjusted kappa >0.90. We resolved differences in interpretation by discussion and added additional emergent themes to the framework. EHM, who has expertise in qualitative analysis, then recoded all transcripts using the revised framework. Further coding was an iterative process grounded in the data. Data were triangulated by comparing the comments of participants within and between groups and analyzed by descriptive and thematic analyses. Survey data were tabulated using SPSS, version 22. We conducted all analyses in 2015.

## Results

In total, 54 men, aged 43–88 years, participated. All were White, non-Hispanic and, reflecting the demographic characteristics of the communities, most were married and had a household income under $75,000 (Table 1). Over one third reported no physical activity outside of their jobs in the past month and about half were current or former smokers, but none reported to be in poor health.

**Table 1:** Participant characteristics (*n* = 54**)**

Age (mean, SD)^a^	62.3 (10.9)
Body mass index (mean, SD)^a^	31.3 (4.6)
	*n* (%)
Household income	
≤ $25,000	10 (18.5)
$25,000-34,999	4 (7.4)
$35,000-49,999	12 (22.2)
$50,000-74,999	11 (20.4)
≥ $75,000	12 (22.2)
Unknown or not reported	5 (9.3)
Marital status	
Now married	41 (75.9)
Separated	1 (1.9)
Divorced	6 (11.1)
Widowed	1 (1.9)
Never married	4 (7.4)
Not reported	1 (1.9)
Employment status^b^	
Employed full-time	11 (20.4)
Employed part-time	5 (9.3)
Self-employed	13 (24.1)
Retired	20 (37.0)
Unemployed (looking for work)	2 (3.7)
Student	1 (1.9)
Unable to work	6 (11.1)
Not reported	1 (1.9)
Race/ethnicity	
White, non-Hispanic	54 (100.0)
Physically active outside of work	
Yes	33 (61.1)
No	20 (37.0)
Not reported	1 (1.9)
Self-reported health status	
Excellent	3 (5.6)
Very good	13 (24.1)
Good	27 (50.9)
Fair	10 (18,5)
Poor	0 (0.0)
Not reported	1 (1.9)
Smoking status	
Current smoker	5 (9.3)
Former smoker	22 (40.7)
Never smoked	26 (48.1)
Not reported	1 (1.9)

^a^Based on available data from 52 participants

^b^Participants could choose more than one option

Findings from the focus groups are divided by research objective. Themes and subthemes that emerged are described in the text and illustrated with quotes.

### Knowledge of modifiable cardiometabolic risk factors

Across the study communities, participants were generally aware of the modifiable risk factors for cardiometabolic disorders, particularly weight status, smoking, and stress. The men described several types of physical activity that were good for their hearts, including walking, mowing the lawn, chopping firewood, sex, and caring for livestock. While some mentioned structured exercises, such as running, fitness classes, or weight lifting, most felt “anything that gets your heart rate up” was beneficial.

Participants in all focus groups identified a number of foods as heart-healthy, particularly fruits, vegetables, whole grains, and lean meats. Locally produced foods, including beef, wild game, and vegetables from home gardens were considered particularly nutritious. However, several men said that they felt confused about which foods are healthy due to conflicting media reports.

“You ever watch the Dr. Oz show? Boy, he’s good. But, God-dang, every other day it’s, ‘This is better, this is better, you know the red, the white, the green, the purple, this fruit, that fruit – you know this [is] where you get your antioxidants, and, you know, this is way too fattening, don’t put any salt, don’t put no sugar, you can get lots better…’ You know, I’m goin’, ‘Oooh, wasn’t it just last week that it was the other way around?’” (Community C, 40–64)

Participants were well-informed about the cardiometabolic risk associated with smoking and emphasized the role of information campaigns and knowing someone with a smoking-related health condition in raising awareness. In four focus groups, men discussed the link between stress and heart attack risk. Self-employment in agriculture was described as demanding and unpredictable, and managing stress was considered critical for maintaining heart health.

“[My dad was] 42 years old when he had his first heart attack. And then he had another one at 43. He was overweight and smoked a pipe all his life. Probably had bacon and eggs most every morning for breakfast… And stress: both times he had a heart attack, it was right in the middle of harvest and, you know, it was a stress-related thing too.” (Community A, 40–64)

### Concern related to heart health and motivation to reduce risk

Despite being knowledgeable of modifiable cardiometabolic risk factors, many participants were fatalistic regarding their risk of a heart attack or stroke, often attributing it to family history or luck. In six focus groups, men shared personal experiences or anecdotes of people they knew who had a cardiac event despite living a healthy lifestyle or of people who had high-risk behaviors and nonetheless enjoyed long, seemingly healthy lives.

“I’m morbidly obese, but when I go in and get a check-up, the doctors all come out and remark that they wish they had my lipid profile… But I have a niece who got left a widow with two small children and her husband was 32 years old when he died, a farmer, died of a heart attack, and no previous evidence of trouble. And here turns out his dad died at 32 years of age… so, you know there was something there, in that, you know, in their genetics or their makeup of their heart that it just didn’t have that many miles in it, and you know bang it’s, it broke.” (Community D, 40–64)

Health was a priority for some but not all participants and concern appeared to be related to age. Several men said that they were motivated to change their behavior following a serious medical event (e.g., heart attack) or disease diagnosis (e.g., diabetes) within their household; they also were motivated by a desire to reduce aging-associated functional decline. Several participants expressed admiration for older men who were capable of strenuous work, particularly chopping their own wood and hunting on foot – activities that were closely associated with fitness and health – and an aspiration to be able to carry out those activities as they aged.

“Ya know, when the doctor said, “Guess what, you’re a diabetic,” then you really don’t have any choice but to change the way you eat, because if you go and eat a big old slice of cheesecake or something, your blood sugar is not going to be under control. Sorry.” (Community F, 40–64)

“I had ‘ta work really hard in losing more weight, because… I had a total knee replacement, and, for my age that I had it at, you know, the average knee lasts maybe 10 to 12 years… and I’m tryin’ to get the most out of my knee.” (Community C, 40–64)

“For me, [my greatest health concern is] just getting old… The things that I do today, I’d like to be able to do when I’m 75 or 80. And that’s what kind of motivates me to try to take care of myself right now, but whether that’s going to pay off, who knows.” (Community A, 40–64)

### Influences on heart-healthy behaviors

#### General influences

Men expressed a great degree of pride in their communities and culture. Popular perceptions of western men as rugged, stubborn, and self-reliant were emphasized and interrelated with thoughts on public health measures. Government involvement in daily activities was generally unpopular. Social norms were represented as fundamentally at odds with health promotion. In fact, many participants boasted about their rejection of healthy behaviors, such as avoiding medical care. Persevering through pain and illness appeared to be venerated and could actually increase a man’s status. For example, in one group, participants expressed respect for a community member who has “got cancer real bad” but is “still getting out in the woods.”

“In different places I worked, you know long after they banned smoking… [they] had a sign in the back [of the] bar, you know, that said ‘Montana, where you could still drink in your car and smoke in the bar.’ And, and that was true for a long time until Montana finally got forced to get on board with the open bottle law. I mean you could never legally drive drunk, but if you weren’t drunk, you could have an open can of beer in the truck out on the prairie or somethin’.” (Community D, 40–64)

“Now I think folks that live like in this kinda country and Montana in general are relatively independent by nature, so if somethin’ does come up it makes you a bit bullheaded, like, ‘I could tough it out.’ And, that’s not the best answer, always, to tough it out.” (Community B, 40–64)

Some men related how, with few chances to meet new people, making a major behavior change could have profound implications on their social and professional lives. For example, men in one group said avoiding bars – a key location for social and business activity in town – could result in isolation and lost income.

“I wanna clean my body out, I wanna quit smoking, ya know, I wanna quit drinking. Well, I mean, to be able to do stuff like that you pretty much have to change your friends at that point in time. And like in this town, who [are] you gonna change your friends to? Ya know, it’s not like you have 900,000 other people that you can go out with and visit with.” (Community F, 40–64)

#### Physical activity influences

Montana’s natural environment was characterized as an outdoor playground with endless opportunities for physical activity in the warm months. Many participants reported enjoying outdoor activities that they believed had a purpose beyond simply leisure, particularly hunting and fishing. However, winter was described as a major barrier to being active. Although men in most communities identified at least one indoor space suitable for physical activity, these often were described unfavorably.

“To improve my health, for me, personally, exercise would be a big one. In the winter… I think all of us are pretty active in the fall and one of the things that attracts us here and keeps us here is hunting and fishing. But, you know, Thanksgiving weekend, that’s all done for the year, and ya get [a] pretty bad case of cabin fever about the end of January…” (Community D, 40–64)

Despite some groups discussing how increasing mechanization on farms made their work less physically demanding, ranching was still juxtaposed with sedentary “desk jobs.” Participants said that they did not have time or interest in participating in structured exercise, which was described by some as a leisure activity for rich, single men, typified as “trust fund guys.” Instead, physical activity was viewed as a natural part of daily routines and could be increased by finding ways to build more activity into their usual schedule, possibly by learning techniques from each other. In one group, two men suggested that self-monitoring using an activity tracker could be helpful.

“I have an app on my smartphone for your pace, you know, steps you do. And I look at that periodically through the day. Because I have a goal I set on that… You know, I’m a farmer and so, you know, instead of just taking the four-wheeler, I just walk, you know, just to get my extra steps or something.” (Community A, 40–64)

Several men said that they disliked structured exercise or did not have the “willpower” to do it. However, getting in shape to participate in preferred activities emerged as a motivation for exercise.

“[Hunting] starts pretty early in the year – unless you want to shoot gophers – and then it starts up again in September, so a lot of us who like to hunt have to get ready for it. So it’s a way of exercising at least.” (Community B, 65+)

Some men also indicated that a group context incorporating an element of competition would be motivating. Men in one group said that working out in a group would encourage them by increasing “accountability to somebody else” and allowing them to “share some ideas” with each other, but would make them feel uncomfortable if the group was not targeted to their fitness level.

“I participated in a working out [group] when they were over here by the school. And, I don’t know, working out… First I forgot that the individuals that I was working out with were a generation or so younger than me and I thought ‘Oh man, I’m working out with all of these good looking women.’ Well, after five minutes into it I didn’t care. All I wanted to do was get the hell out of there and not die or throw up. (Group laughs.) So, it would be kind of interesting to have, I don’t know, some kind of organization for folks in our general shape, in our age bracket, you know, without a drill-master with a whip on us to give us some direction on how to properly work out and so forth and so on.” (Community A, 40–64)

Living out of town emerged as a barrier to participation to group-based physical activity. Many men who lived outside of town did not want to travel to town to participate in something on a regular schedule. Participants in one group suggested that men might be more inclined to take part in a program that met less frequently, but integrated at-home activities that couples could do together.

“A lot of us live out of town, so the idea of coming back to town twice a week isn’t really appealing, unless there was a program that could be designed that is compatible for a man and wife to kinda try to do together. That might work for a big part of our community, ‘cuz of the distance and inconvenience of coming back and forth to town or something.” (Community A, 40–64)

#### Healthy eating influences

Participants often indicated a preference for calorie-dense, animal-source foods and expressed the opinion that “healthy” foods are less tasty. For instance, in describing a heart-healthy diet, men in three focus groups cited the adage, “if it tastes good, spit it out.” Participants considered meat, generally accompanied by potatoes, to be the most important meal component. While some articulated the benefits of increasing consumption of fruits and vegetables, this generally did not imply a shift away from meat-based meals. In addition, healthy eating was associated with giving up favored foods and more laborious food preparation.

“A steady diet of just beef is not good, but a vegetarian diet is not living life.” (Community C, 40–64)

Hunting and fishing were described as major sources of meat and, in four groups, men reported that they or their wives were involved with growing their own vegetables and fruits during the summer months. However, local grocery and restaurant options for fresh produce were described as limited, overpriced, and of poor quality.

“My wife gardens. You know, we eat a lot of fresh vegetables just because we would rather grow our own if we can, so we know where it came from.” (Community A, 40–64)

“Sometimes the lack of choice at the local market can be kind of challenging. [The store owner] knows the market real well, so I don’t blame them for not putting things on the shelf that are going to rot and they end up throwing them away.” (Community A, 40–64)

Some men felt that healthy eating in a social context was challenging, because of few healthy choices and the social expectation that they would accept food that was offered to them.

“Like I said, you know, my wife. I always said that. I mean, she does the grocery shopping. I mean, she makes my lunch every day and makes breakfast and kind of, you know, I don’t say much, ‘cuz she could say, ‘Well, if you don’t like it, make it yourself.’” (Community A, 40–64)

Cooking was described as a highly gendered activity carried out almost exclusively by women. However, participants in two groups indicated that they enjoyed cooking and would be interested in taking cooking classes.

Some men reported participating in a state-wide dietary behavior change program and said that they found it to be helpful in changing eating habits through its emphasis on tracking and self-monitoring total fat intake. One participant specified that, to be credible, dietary educators should be “bigger around than my little finger.”

“In terms of healthy eating, I went through the [Healthy Lifestyles Program]… and I find that [it] did stick with me and it was very simple. All I had to do was count grams of fat and keep them to a pre-set number based on my size, blah, blah, blah. And it worked… I’m still monitoring fat intake basically to this day and that was probably two years ago, or at least for me, it was probably two years ago. So, that definitely made a difference. And it’s not that easy to just cut fat out of your diet. It’s the good stuff, it’s the good-tasting stuff.” (Community A, 40–64)

#### Tobacco use influences

Men described tobacco use as common in their communities and associated chewing tobacco with ranching. Although most felt rates of smokeless tobacco (e.g., chewing tobacco) use remained high, participants agreed that smoking had declined in recent years and attributed this shift to increased awareness and the state-wide indoor smoking ban. However, a few participants suggested that tobacco use may be rising among youth.

“[In] the ranching community, there’s a lotta chewers, I think. Just,’cuz, when you’re out in the wind in this country, you can’t light up a cigarette, you know, and smoke. Or when you’re handling hay, you’re workin’ in the barn, you can’t smoke, but you can chew…” (Community D, 40–64)

Many men talked about the addictive properties of tobacco products and detailed their own struggles or those of their friends and family in quitting smoking or chewing. Quitting smoking was framed around an individual’s “willpower,” although living or socializing with smokers were acknowledged to be barriers.

“I quit smoking when I was 21 years old. The problem was not the quitting smoking, it was the friends that still smoked, ‘cuz [when] you get around people that do smoke when you’re trying to quit, it’s very hard.” (Community B, 65+)

“Four or five years after I quit smoking, the salesman come and was talking insurances and stuff. He handed me a cigarette, I had it in my mouth, and he’d lit his, and he’d started to reach over there for mine, and I thought, “What!” I pulled that cigarette out of my mouth and it was just as normal as the day I quit smoking to get that thing and oh boy. (Laughs.)” (Community E, 65+)

Several participants said that it was common for men in their communities to start using smokeless tobacco as a strategy to quit smoking, or to start smoking to quit chewing. Some participants also talked about eating a lot of candy after giving up smoking.

“I started smokin’ to quit chewin.’ It helped me quit chewin’. I’ve been chewin’ since I was 12 years old.” (Community D, 40–64)

## Discussion

**Fig. 2:**
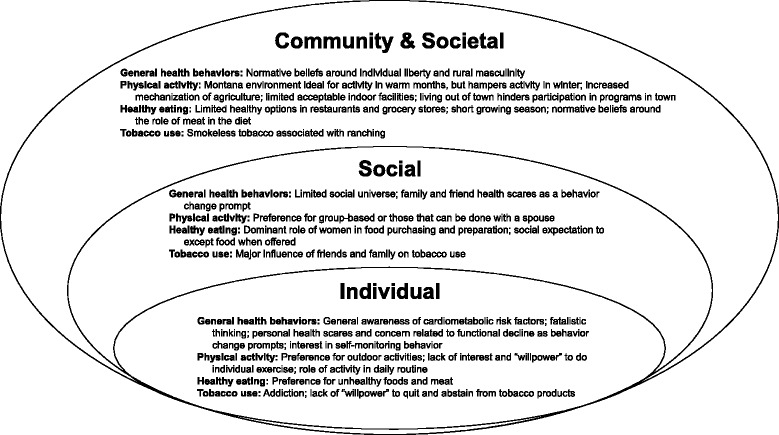
Socioecological model depicting influences on cardiometabolic risk behaviors for rural men in this study. This figure depicts factors at multiple levels of the socioecological model influencing cardiometabolic risk behaviors among rural men in this study

This study examined the factors that influence physical activity, diet, and tobacco use behaviors in rural men, a group at elevated risk for cardiometabolic disorders. To our knowledge, this study is the first in-depth exploration of knowledge, concerns, and influences on heart-healthy behaviors for rural men in the western U.S. The results suggest this population is generally knowledgeable about modifiable cardiometabolic risk factors, but skeptical about the degree to which risk could be reduced by adopting healthy behaviors and confronted by considerable barriers to taking action. We believe these findings are relevant not only to rural men in Montana, but also to those in the bordering states of Idaho, North Dakota, South Dakota, and Wyoming, where demographic characteristics are similar [23], large proportions of residents live in rural areas [21] and agriculture remains critical to rural economies [23]. Differences were not observed between men in the midlife focus groups and men in the older-aged groups, probably because age variation was modest, with most participants aged 50 to 70 years. Figure 2 depicts factors at multiple levels of the socioecological model influencing behaviors that may affect cardiometabolic health among rural men. By identifying specific factors and their level of influence, this research highlights potential targets for intervention.

Overall, strong normative beliefs around rural masculinity clashed with public health goals. Rural men were characterized as “tough guys,” who work with their hands, hunt their own food, struggle against the odds to keep their farms and ranches viable, and consider seeking help a sign of weakness. Consistent with previous research [26, 27], an underpinning theme was that masculine men have more important things do to than to obsess over their health or bodies. These findings support and extend existing research linking normative rural masculinity and the adoption of risky or unhealthy behaviors [28]. A major health concern among men in this study was that they would develop a condition that would incapacitate them and limit self-reliance. This suggests that health messages that focus on cardiovascular disease and diabetes as disabling illnesses may help persuade rural men to adopt healthy behaviors by leveraging cultural ideals of masculinity.

We found participants to oppose being told what to do and to resist restrictions on their behavior, but open to making changes if those changes were on their own terms. For instance, tracking and self-monitoring diet and activity were considered acceptable and appear to have been helpful to participants who have trialed them. Health was considered an individual responsibility and behavior change was perceived to be feasible by anyone with sufficient motivation. These sentiments should be taken into account when designing interventions targeted at rural men. However, participants’ acknowledgement of the critical role of their social environment in influencing health behaviors suggests a need to also engage rural men’s friends and family in promoted activities. Contrary to previous research with rural Latino and African American men [19, 20], participants in this study did not report churches or church leaders as an important influence on health behaviors.

Despite increasing mechanization in agriculture, participants generally felt that they had sufficient opportunities to build physical activity into their daily routines. They overwhelmingly preferred to get their exercise from outdoor activities – particularly hunting and fishing – and cited winter as a major barrier to being active year-round. Group-based exercise was perceived more favorably than individual activity, in part because it provided scope for competition and social accountability. However, participants stressed that they would not take part if they felt uncomfortable or if frequent travel was required. Messaging focused on preparing your body to be in optimal shape for hunting or to participate in a team sport may be appropriate and effective for this population.

Taste preferences and social norms around meat consumption emerged as major barriers to dietary behavior change. The popular perception that men need meat to complete a meal [29–31], was firmly held by men in this study. Raising and hunting animals for meat was central to participants’ identities and they strongly objected to the idea that meat consumption should be limited. Given these beliefs and attitudes, health promotion strategies that emphasize reducing consumption of meat and other animal products are unlikely to be successful. An alternative approach could be highlighting the benefits of fruit and non-starchy vegetable intake, with a focus on locally-available produce. As widely documented by others [29, 32], we found food-related activities to be highly gendered, with women largely in control of food content and preparation. This suggests a need to include both genders in dietary intervention activities, even if the primary target group is rural men.

Men in this study clearly understood the benefits of avoiding tobacco and perceived smoking cessation to be socially desirable. However, many doubted that they could stop smoking and successfully abstain from tobacco, especially when continuing to socialize with tobacco users. Although prevalence of smoking has dropped substantially in recent years, several states continue to have high rates of smokeless tobacco use, including Montana and each of its bordering states [33]. In fact, in Montana, prevalence of smokeless tobacco use increased by 12.7 % between 2011 and 2013, nearly paralleling the decline in cigarette smoking. This suggests a need for strategies specifically targeted at helping men develop the self-efficacy to quit smoking or chewing without switching to another form of tobacco or to candy.

Our findings must be interpreted in light of several limitations. While men were recruited from the community, they self-selected to participate, and thus may not be representative of all overweight, sedentary rural men in terms of their understanding of and ideas related to cardiometabolic health. In addition, the screening tool relied on self-report measures of weight, height, and physical activity, and research suggests men tend to under-report weight and over-report height [34] and adults tend to overestimate physical activity [35]. Within the groups, men’s contributions may have been influenced by social-desirability pressures that made them feel the need to promote their masculinity, especially given the small and tight-knit nature of the communities. Further, the facilitator and research team were all female and this may have influenced participant responses, coding, and interpretation. However, the sample was purposive and resembled the population in the geographic area of interest, and the use of the same facilitator for all focus group discussions strengthened measurement consistency. Finally, collection of the data in September and October – when fresh foods are readily available and the weather is mild – may have also influenced participants’ responses.

## Conclusions

The results of this study suggest that individual preferences, normative beliefs related to masculinity, and limited access to healthy food and activity options during the winter are important determinants of health behaviors in rural men in the western U.S. These findings provide guidance for possible intervention strategies to promote cardiometabolic health in this population. Future public health interventions could leverage messages that resonate with rural men, such as the benefits of healthy behaviors to maintaining physical function, foster family and peer support for positive behavior change, and support policies and programs that improve the accessibility and affordability of healthy foods and group exercise classes for men. For example, low-cost exercise sessions that focus on self-monitoring and keeping one’s body in shape for hunting and working outside may be of interest to this population. Overall, more intensive efforts are needed to engage men, particularly rural men, in chronic disease prevention interventions, and to design programs that suit their needs and preferences.

### Availability of data and materials

Due to the small size of the communities where we conducted the focus groups and ethical considerations related to participant confidentiality, it is not possible to make data from this study publically available.

## Abbreviations

NIFA: National Institute of Food and Agriculture
U.S.: United States
